# Celiac Crisis Complicated by Refeeding Syndrome: A Case Report and Pediatric-Adapted Diagnostic Criteria

**DOI:** 10.3390/reports9010072

**Published:** 2026-02-28

**Authors:** Noemi Zampatti, Federico Medina, Angela Calvi, Federica Malerba, Marco Crocco

**Affiliations:** 1 Department of Neuroscience, Rehabilitation, Ophthalmology, Genetics, Maternal and Child Health, University of Genoa, 16132 Genoa, Italy; 5283390@studenti.unige.it (N.Z.); federicamalerba@gaslini.org (F.M.); 2 Division of Pediatrics, Department of Health Sciences, University of Piemonte Orientale, 28100 Novara, Italy; 20011805@studenti.uniupo.it; 3 Independent Researcher, 16100 Genoa, Italy; calvi.angela58@gmail.com; 4 UOC Pediatria e Neonatologia Imperia, IRCCS Istituto Giannina Gaslini, 18100 Imperia, Italy; 5 Pediatric Gastroenterology and Endoscopy Unit, IRCCS Istituto Giannina Gaslini, 16147 Genoa, Italy

**Keywords:** celiac disease, celiac sprue, gluten-free diet, intussusception, malnutrition, metabolic disorder

## Abstract

**Background and Clinical Significance**: Celiac disease (CD) is a gluten-triggered immune enteropathy that may rarely present as Celiac crisis (CC), a life-threatening condition marked by severe diarrhea, dehydration, metabolic derangements, and acute malnutrition. Pediatric diagnostic criteria are lacking, and despite its reduced incidence in high-income countries, CC remains a critical complication, potentially associated with refeeding syndrome. **Case Presentation**: We report the case of a 23-month-old girl presenting with chronic diarrhea, weight loss, iron-deficiency anemia, hypoalbuminemia, and coagulation abnormalities. Serology confirmed CD, and a gluten-free diet (GFD) was initiated. However, the patient experienced clinical deterioration consistent with CC. Her course was further complicated by refeeding syndrome, ileo-ileal intussusception, and deep vein thrombosis, requiring corticosteroids, anticoagulation, and multidisciplinary nutritional support. Full clinical recovery was achieved within two months. **Conclusions**: This case highlights the life-threatening potential of CC and the necessity for early recognition. Timely GFD initiation, correction of metabolic abnormalities, and monitoring for refeeding syndrome are essential. We propose pediatric-adapted diagnostic criteria to facilitate earlier recognition and standardize the management of CC. The proposed framework includes major and minor criteria based on the rapid onset of gastrointestinal symptoms with serological evidence of CD autoimmunity, accompanied by clinical instability requiring hospitalization or intensive support and multiple indicators of systemic compromise.

## 1. Introduction and Clinical Significance

Celiac disease (CD) is a chronic immune-mediated enteropathy precipitated by the ingestion of gluten in genetically predisposed individuals [1]. It exhibits a broad clinical spectrum, ranging from silent or subclinical forms to severe malabsorptive syndromes involving both gastrointestinal and extraintestinal systems [1]. The global prevalence of CD is estimated at 1–2%, with an upward trend in recent years attributed to increased awareness, improved screening protocols, and a general rise in autoimmune conditions [2].

To date, the only effective treatment for CD remains a strict, lifelong gluten-free diet (GFD), which typically results in clinical remission within a few months [3]. However, in rare circumstances, particularly in young children, CD can present as Celiac Crisis (CC), a fulminant and potentially life-threatening complication. CC is now rare in high-income countries due to earlier diagnosis and implementation of screening programs. Nonetheless, when it occurs, it poses significant diagnostic and therapeutic challenges [4].

The CC is characterized by profuse diarrhea, severe dehydration, electrolyte imbalances, metabolic acidosis, and acute nutritional deterioration [5]. Infectious triggers and recent surgical procedures have been identified as potential precipitating factors [6]. No diagnostic criteria are available for pediatric age.

Refeeding syndrome is a rare, but potentially serious complication of CC following the initiation of a GFD. It arises from abrupt metabolic shifts and intracellular electrolyte depletion, a risk markedly increased in CC due to chronic diarrhea, protein–energy malnutrition, and rapid nutritional repletion, which together aggravate fluid and electrolyte losses [7,8]. Complications may also include severe micro- and macro-nutrient deficiencies, coagulopathy, dysmotility, and thrombotic events, all of which necessitate a multidisciplinary management approach [9,10,11,12,13].

We present the case of a 23-month-old girl who developed CC with severe multisystem involvement. Her clinical course was complicated by refeeding syndrome, ileo-ileal intussusception, coagulopathy with deep vein thrombosis (DVT), requiring complex therapeutic interventions. By detailing the multidisciplinary approach and presenting the adapted diagnostic criteria, we aim to streamline early diagnosis and improve outcomes in pediatric CC.

## 2. Case Presentation

A 23-month-old female patient presented with chronic diarrhea. She was initially admitted to a secondary hospital for investigation, where laboratory tests revealed anemia (Hb 9.7 g/dL). She was discharged in good general clinical condition with a diagnosis of acute gastroenteritis and moderate dehydration.

The patient was born at term (39 weeks gestational age) with a birth weight of 2480 g (5th percentile, small for gestational age). Neonatal adaptation was normal. Complementary feeding was initiated at 5 months, with gluten introduced between the 5th and 6th months. Family history was negative for gastrointestinal, autoimmune, cardiovascular, or hematologic disorders.

Due to chronic diarrhea associated with significant weight loss (−30% since symptom onset) and fatigue, the patient was re-evaluated in the emergency department 10 days later. Blood tests confirmed iron-deficiency anemia and revealed a weakly positive CD screening (IgA anti-tTG antibodies 2× ULN, EMA positive, IgA anti-DGP antibodies 2× ULN) and hypothyroidism. Stool cultures and parasitology were negative. An abdominal ultrasound was unremarkable.

Given the clinically stable condition and the low-titer IgA anti-tTG antibodies, an esophagogastroduodenoscopy (EGD) was scheduled 40 days later, according to ESPGHAN guidelines [3]. Pre-procedural blood tests revealed Hb 9 g/dL, PLT 772,000/mm^3^, and significant coagulation abnormalities (PT ratio 2.49, PTT ratio 1.63), which were confirmed on repeat testing (PT ratio 3.31, PTT ratio 1.86). Due to severe coagulopathy, the endoscopic procedure was suspended. A femoral venous access was placed for plasma transfusion and vitamin K administration. Shortly after catheter placement, the patient developed thrombophlebitis of the left lower limb with marked swelling, prompting the initiation of anticoagulation therapy with enoxaparin (100 IU/kg twice daily).

A diagnosis of CC was established according to current diagnostic criteria used in pediatric age [9]. Specifically, the patient presented acute clinical manifestations, including severe dehydration; irritability; abdominal pain; hypoalbuminemia; electrolyte imbalance; growth deceleration; and metabolic instability; requiring hospitalization and systemic corticosteroid therapy.

Celiac serology was repeated prior to initiating a gluten-free diet (GFD) and demonstrated marked positivity (IgG anti-DGP antibodies 19× ULN, EMA positive, IgA anti-tTG antibodies 20× ULN), thereby supporting and confirming, according to ESPGHAN guidelines [3], the clinical diagnosis of CC in the absence of histological evaluation, which was contraindicated due to coagulopathy.

A GFD was initiated. Despite slow initial clinical improvement, including the resolution of diarrhea, the patient experienced critical clinical deterioration two weeks after starting the GFD. Symptoms included persistent abdominal pain, irritability, constipation, asthenia, hyperphagia, and frequent nocturnal awakenings due to hunger. Given this deterioration, the family decided to transfer the patient to our regional referral center for CD.

**Figure 1:**
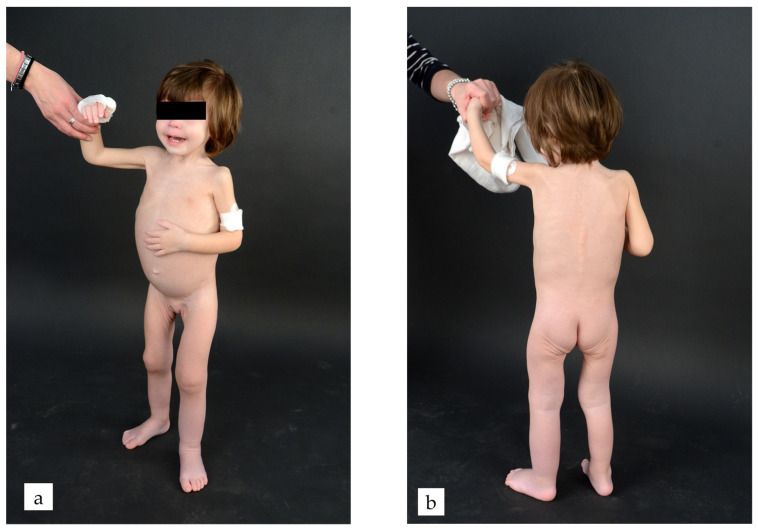
(**a**) Severely dystrophic state with distended abdomen; (**b**) “Tobacco pouch” appearance of buttocks indicating muscle wasting.

Upon admission, the patient appeared to be in poor general condition (Figure 1): irritable, pale, with sunken eyes, inconsolable crying, and a severely dystrophic state. The abdomen was markedly distended and tense. The left lower limb was diffusely swollen with shiny skin. Weight was 10.3 kg (<5th percentile, according to CDC growth charts), and height was 82.5 cm (10th–25th percentile, according to CDC growth charts).

Laboratory findings revealed microcytic hypochromic anemia, thrombocytosis, hypoalbuminemia, elevated transaminases, severe iron deficiency, severe dyslipidemia, and multiple vitamin deficiencies (Table 1). TSH was increased with normal FT4 and negative anti-thyroglobulin and anti-thyroid peroxidase antibodies. IgA anti-tTG antibodies were >200 U/mL (normal value < 20 U/mL). Thrombophilia screening and C-reactive protein (CRP) were negative.

**Table 1:** Laboratory findings at admission.

Parameter	Value	Reference Range
Hemoglobin (Hb)	8.1 g/dL	11.0–13.5 g/dL
Mean Corpuscular Volume (MCV)	66.2 fL	75–86 fL
Mean Corpuscular Hemoglobin (MCH)	17.8 pg	27–32 pg
Platelets (PLT)	712,000/mm^3^	150,000–450,000/mm^3^
Albumin	2.78 g/dL	3.8–5.0 g/dL
AST	65 U/L	<35 U/L
ALT	70 U/L	<35 U/L
GGT	51 U/L	8–30 U/L
Ferritin	<5 ng/mL	12–200 ng/mL
Total Cholesterol	304 mg/dL	<170 mg/dL
Triglycerides	288 mg/dL	<150 mg/dL
Vitamin D (25-OH)	4.5 ng/mL	20–50 ng/mL
Vitamin A	14 µg/dL	20–43 µg/dL
Vitamin E	209 µg/dL	500–1800 µg/dL
TSH	6.18 µU/mL	0.2–4.2 µU/mL
IgA anti-tTG	>200 U/mL	<20 U/mL

The clinical and laboratory picture was consistent with CC and refeeding syndrome. Table 2 illustrates the differential diagnosis considered.

The GFD was continued with a restricted intake regimen (starting with 50% of daily caloric requirements recommended for age) and increasing oral intake gradually [14] and strict control of biochemical parameters. Oral corticosteroid therapy (betamethasone 0.1 mg/kg/day) was initiated for the treatment of CC but was rapidly tapered and discontinued after a 10-day course. Parenteral nutrition was omitted to reduce the risk of refeeding syndrome. Micronutrient deficiencies were addressed with intravenous iron infusion (ferric carboxymaltose 15 mg/kg) and daily oral supplementation of multivitamin and multimineral dietary supplement (Folic acid: 130 mcg, Pantothenic acid: 6 mg, Vitamin A: 320 mcg, Biotin: 24 mcg, Vitamin B1 (thiamine): 0.7 mg, Vitamin B2 (riboflavin): 1.1 mg, Vitamin B6: 0.7 mg, Vitamin B12: 1 mcg, Vitamin C: 45 mg, Vitamin D: 8 mcg, Vitamin E: 3.6 mg, Vitamin K: 30 mcg, Niacin: 11 mg, Calcium: 240 mg, Iron: 6.8 mg, Phosphorus: 242 mg, Iodine: 90 mcg, Magnesium: 80 mg, Manganese: 1 mg, Copper: 0.4 mg, Selenium: 15 mcg, Zinc: 6 mg.

**Figure 2:**
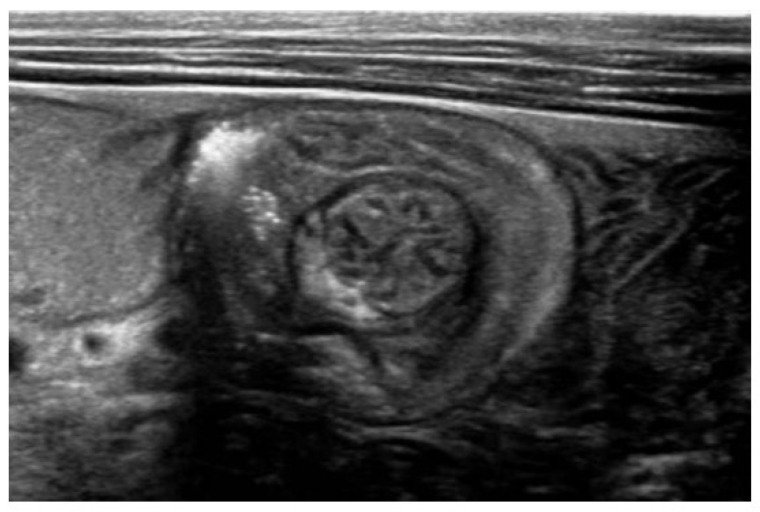
Target sign on abdominal ultrasound indicating intussusception.

**Figure 3:**
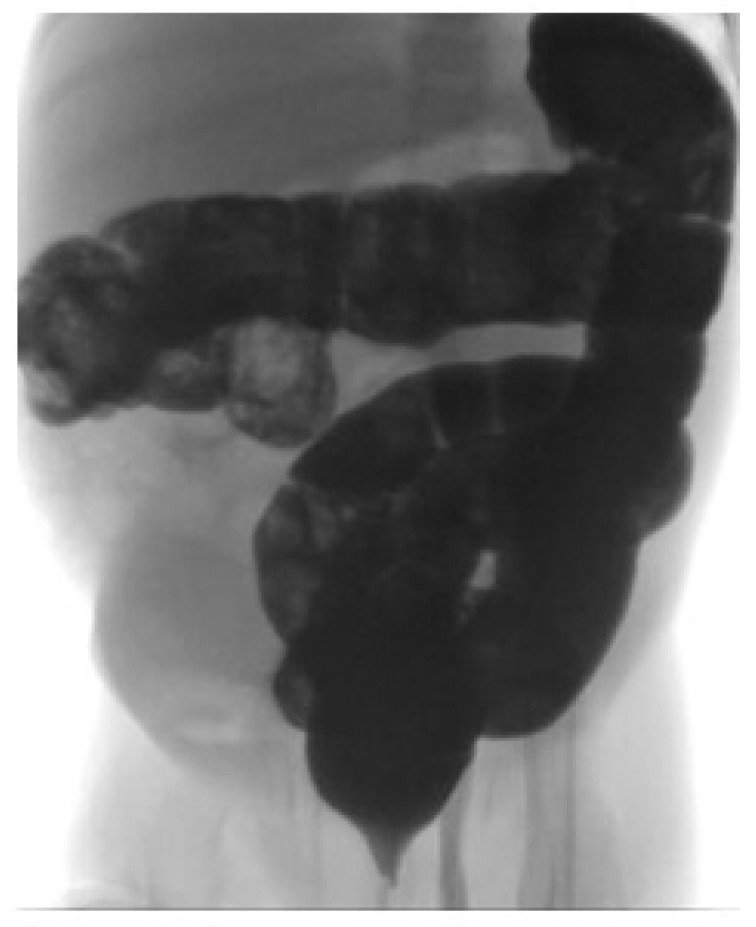
Contrast enema performed for reduction in intussusception.

Persistent abdominal pain prompted an abdominal ultrasonography, which revealed an ileo-ileal intussusception with the classic target sign (Figure 2). A contrast enema was performed (Figure 3), resulting in the resolution of the intussusception, confirmed by a follow-up abdominal CT.

Persistent swelling of the left lower limb warranted a color Doppler ultrasound, which demonstrated thickening of the common femoral vein wall and thrombotic apposition at the distal segment of the external iliac vein. Enoxaparin therapy was continued for a planned duration of 3 months.

**Figure 4:**
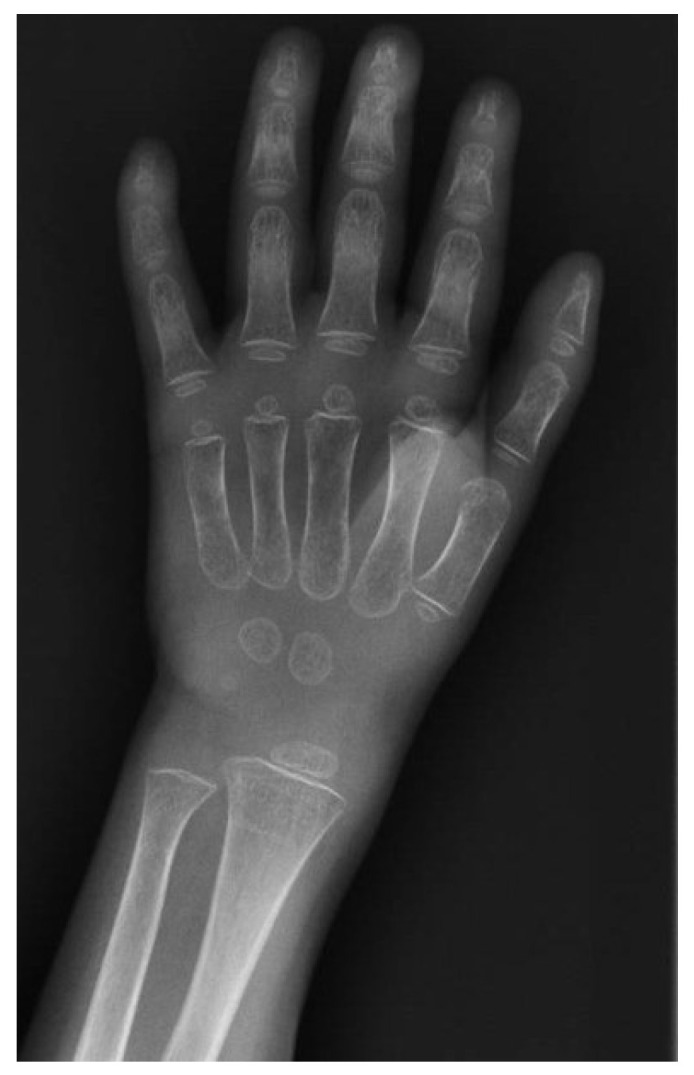
Wrist X-ray showing severe bone demineralization.

A wrist X-ray showed severe bone demineralization with submetaphyseal resorption (Figure 4), consistent with intestinal malabsorption, in the absence of overt radiological signs of rickets. Vitamin D supplementation was maintained for 6 months.

**Figure 5:**
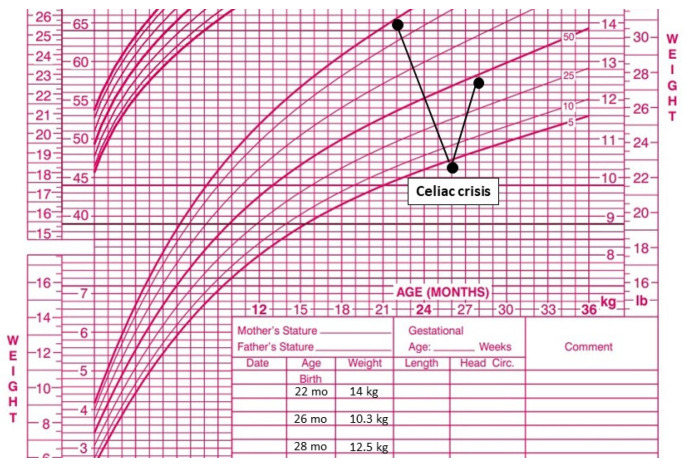
Weight progression over time.

Throughout hospitalization, the patient’s oral intake slowly increased, and progressive improvement in nutritional status was observed. At the 2-month follow-up after discharge, the patient was in good general condition and strictly adherent to the GFD. She had gained 2.2 kg since discharge (Figure 5), with normalized bowel habits, resolution of clinical and laboratory anomalies.

## 3. Discussion

CC is a rare complication of CD, predominantly affecting the pediatric population at onset. The exact incidence is unknown, with evidence limited to small case series. Despite its potential severity, CC remains under recognized and is not defined as a distinct entity in current leading guidelines (American College of Gastroenterology; European Society Paediatric Gastroenterology, Hepatology and Nutrition; British Society of Gastroenterology [3,15,16]).

While diagnostic criteria for CC in pediatric patients are not universally standardized, the literature consistently describes it as an acute, life-threatening complication characterized by the rapid progression of gastrointestinal symptoms, severe metabolic and electrolyte disturbances, malnutrition/dehydration, and the need for hospitalization or parenteral nutrition. In the first case series published in 1953, Andersen and Di Sant’Agnese reported a case fatality rate of 9% among 35 children with CC [17,18]. Although mortality and morbidity have decreased significantly since then, CC remains largely underdiagnosed. Trovato et al. reported a prevalence of 1.7% in new-onset CD [19], whereas a recent meta-analysis identified only 195 reported cases (both children and adults), with the majority drawn from a single study. This meta-analysis reported two deaths during the acute phase: a 2.5-year-old girl due to metabolic derangements and a 28-year-old female due to refeeding syndrome. Nine patients required intensive care [20].

In our case, the initial delay in recognizing CC and referring the patient to a specialized center may have contributed to clinical deterioration and increased the risk of refeeding syndrome. A specialized “hub and spoke” referral system improves the management of such complex cases, as standardizing diagnostic and therapeutic pathways reduces clinical risk [21].

Currently, there are no validated diagnostic criteria for the pediatric age group. The criteria proposed by Jamma et al., developed through expert consensus, have not been formally validated [22]. The absence of specific pediatric criteria causes diagnostic delays and hinders the comparison of different studies. We applied modified criteria based on Jamma et al. [22], adapting them to the pediatric population and including the most frequent signs or symptoms identified by recent literature [20]. We also specified the necessity of positive CD screening (including Point-of-Care Testing) or histological diagnosis. This addition is based on evidence that in 183 patients with CC, 11 had normal tTG IgA levels, while EMA testing was more sensitive (positive in all 20 tested) [20]. Although anti-DGP IgG results were not reported in those patients, they may be useful when tTG IgA is negative or low, as anti-DGP IgG may rise before seroconversion of anti-tTG IgA [23]. The proposed diagnostic criteria for pediatric CC are detailed in Table 3.

In our patient, tTG IgA levels were initially low despite severe symptoms, consistent with population-based studies showing a lack of correlation between seropositivity levels and symptom severity [28]. We emphasize the need to perform CD screening in all patients with symptoms compatible with CC and to repeat it if the clinical condition worsens.

In children with high-titer anti-tTG IgA (>10× ULN) confirmed by positive EMA, a diagnosis of CD can be established without upper endoscopy, according to ESPGHAN guidelines [3]. This non-biopsy approach is particularly relevant in the context of severe clinical presentation, where endoscopic procedures may be unsafe or contraindicated due to severe coagulopathy, electrolyte imbalances, or hemodynamic instability. Therefore, the diagnosis of CC should remain primarily clinical, supported by serological findings, with histological evaluation reserved for clinically stable patients or those with atypical presentations.

The indication for endoscopic examination, in a patient with CC without ESPGHAN criteria for non-biopsy approach, should be discussed multidisciplinarily, balancing clinical utility against procedural risk. Current clinical guidelines do not address CC management; consequently, most patients undergo endoscopy, up to 95% in a recent systematic review of Dhali et al. [20]. However, we disagree with the conclusion of Dhali et al.: “histopathological analysis is still crucial for confirming the diagnosis of CD” in this context. In our opinion, the diagnosis of CC remains clinical, supported by serology, while histology should be the exception rather than the rule in critical patients. In our case, biopsy was contraindicated due to coagulopathy, highlighting the relevance of non-invasive diagnostic strategies.

We used oral corticosteroids while avoiding parenteral nutrition to minimize the risk of electrolyte shifts. The use of corticosteroids in CC has been historically described as a rescue therapy aimed at rapidly controlling severe intestinal inflammation and restoring absorptive function [29]. However, their use remains limited; a recent meta-analysis noted only 8.2% of patients received them [20]. Corticosteroids are suggested in refractory or severe inflammation [30,31], potentially restoring brush border enzymatic activity and reducing mucosal inflammation [29,32,33,34]. In the context of CC, corticosteroids may be considered in selected severe cases characterized by marked inflammation or inadequate response to GFD and supportive therapy alone. However, caution is warranted in patients with hypokalemia and refeeding syndrome, as steroids may exacerbate electrolyte imbalances [9]. Furthermore, as infections are potential precipitants of CC, steroids should be avoided if sepsis is suspected [35]. To mitigate systemic side effects, there is increasing interest in locally active corticosteroids like budesonide, which has shown efficacy similar to systemic steroids [36,37], however capsule formulations may limit use in younger children.

Our patient’s coagulopathy and subsequent DVT underscore the fragile hemostatic balance in severely malnourished CD patients. Although thrombosis is not a classical manifestation of CD, it can occur due to a combination of dehydration, systemic inflammation, deficiencies in vitamin K, folate, and vitamin B12, leading to endothelial dysfunction and hypercoagulability [10,11,12,38]. Additionally, the patient developed ileo-ileal intussusception, a complication described in 1.2–25% of new-onset CD cases. In CD, chronic inflammation and malabsorption lead to mucosal atrophy, bowel wall edema, altered motility, lymphoid hyperplasia, which predispose to intussusception [39,40,41].

The clinical deterioration following the initiation of GFD raised the suspicion of refeeding syndrome, a potentially lethal complication of refeeding in severely malnourished patients [42]. Our patient exhibited multiple risk factors, including severe weight loss, hypoalbuminemia, and baseline electrolyte depletion. A structured preventive strategy was therefore adopted: caloric intake was initiated at approximately 50% of age-recommended requirements, parenteral nutrition was avoided, biochemical parameters were closely monitored, and early vitamin and micronutrient supplementation was provided. Consistent with ASPEN consensus recommendations [8] and recent pediatric literature [43], this cautious approach ensured metabolic stability during the early phase of GFD initiation and was associated with a favorable clinical outcome.

In summary, CC creates a clinical milieu of severe malnutrition, mucosal injury, electrolyte derangements, and coagulopathy, predisposing to refeeding syndrome upon nutritional repletion, coagulopathy from vitamin K deficiency, and intussusception from altered bowel motility and mucosal changes. These complications are interrelated, reflect the severity of the underlying disease, and require coordinated management to prevent morbidity and mortality.

The clinical challenges presented by this case highlight several critical research gaps regarding CC. Currently, the lack of standardized pediatric diagnostic criteria and consensus definitions hinders timely diagnosis and accurate epidemiological reporting. Our proposed diagnostic criteria for pediatric CC are a pragmatic clinical tool derived from the adult consensus criteria [18], integrated with the most frequently reported clinical features in the literature [20]. The proposed criteria should be considered exploratory and require validation through multicenter studies and expert consensus processes before widespread adoption. Existing data are confined to a limited number of case series, reflecting a significant gap in translational research concerning the pathophysiology of CC. Consequently, there is a limited understanding of specific risk factors and optimal therapeutic strategies. While a strict GFD is universally recommended, the role, timing, and dosing of adjunctive supportive therapies—including oral supplementation, parenteral nutrition, and immunomodulators—remain poorly defined. The use of corticosteroids is largely empirical, based on management strategies for other chronic autoimmune intestinal diseases; however, the risk of refeeding syndrome is significantly higher in CC and may be precipitated by either systemic corticosteroid treatment or parenteral nutrition. Furthermore, the limited characterization of both acute and long-term outcomes currently precludes robust risk stratification for subsequent complications.

## 4. Conclusions

Celiac crisis is a rare but life-threatening complication of CD. This case report illustrates the severe multisystemic nature of CC, reinforcing the importance of early diagnosis and a multidisciplinary approach. Comprehensive supportive care and close monitoring for complications, including refeeding syndrome and thrombosis, are essential.

To bridge the current research gap and reduce diagnostic delays, we propose standardized diagnostic criteria adapted for the pediatric population. This framework establishes a diagnosis based on the presence of at least one major and three minor clinical criteria. Further research is needed to better understand the risk factors and optimal management of this severe condition.

## Figures and Tables

**Table 2:** Differential diagnosis of celiac crisis [5,7].

Infections	viral gastroenteritis, post-infectious gastropathy, tropical sprue, bacterial overgrowth syndrome, AIDS enteropathy, Whipple disease, parasitic infestation, Helicobacter pylori-positive gastritis and peptic duodenitis
Drugs	non-steroidal anti-inflammatory drugs, antineoplastic agents, immune modulatory drugs, angiotensin receptor blockers
Immune-inflammatory conditions	autoimmune enteropathy, Crohn’s disease, ulcerative colitis associated duodenitis, eosinophilic gastroenteritis, food protein sensitive enteropathies (allergies to chicken, cow’s milk, eggs, fish, soy), collagenous sprue, immunodeficiencies (including common variable immunodeficiency)
Others:	pancreatic insufficiency, laxative use, intestinal lymphoma

**Table 3:** Proposed celiac crisis criteria adapted for pediatric age [20,22].

Acute onset or rapid progression of gastrointestinal symptoms attributable to CD (positive CD screening (including Point-of-Care Testing) or histological diagnosis (Marsh Oberhuber ≥ 2 [24]) associated with:
**At least 1 of the main criteria:**
Need for hospitalization
Need for parenteral nutrition
Need for systemic corticosteroid
**At least 3 of the minor criteria:**
1. Severe dehydration, fluid deficit of ≥10% of body weight [25]
2. Neurologic dysfunction including irritability and changes in behavior
3. Renal dysfunction, urine output <0.5 mL/kg/hour for ≥6 h, Initiation of renal replacement therapy or fluid overload ≥ 20% [26]
4. Metabolic acidosis, pH < 7.35
5. Hypoproteinemia, serum albumin < 3.4 g/dL for those ≥7 months and <2.5 g/dL for those <7 months [27]
6. Abnormal electrolyte levels including hypernatremia/hyponatremia, hypocalcemia, hypokalemia, or hypomagnesemia
7. Decanalization of the growth curve, crossing downward of at least two major percentile lines
8. Abdominal pain or vomiting

## Data Availability

The data presented in this study is available on request from the corresponding author.

## Abbreviations

The following abbreviations are used in this manuscript:

ALT	Alanine Aminotransferase
ASPEN	American Society for Parenteral and Enteral Nutrition
AST	Aspartate Aminotransferase
CC	Celiac Crisis
CD	Celiac Disease
CRP	C-reactive Protein
CT	Computed Tomography
DGP	Deamidated Gliadin Peptide
DVT	Deep Vein Thrombosis
EGD	Esophagogastroduodenoscopy
EMA	Endomysial Antibodies
ESPGHAN	European Society of Pediatric Gastroenterology, Hepatology and Nutrition
FT4	Free Thyroxine
GFD	Gluten-Free Diet
GGT	Gamma-Glutamyl Transferase
Hb	Hemoglobin
MCH	Mean Corpuscular Hemoglobin
MCV	Mean Corpuscular Volume
PLT	Platelets
PT	Prothrombin Time
PTT	Partial Thromboplastin Time
SGA	Small for Gestational Age
TSH	Thyroid-Stimulating Hormone
tTG	Tissue Transglutaminase
ULN	Upper Limit of Normal
